# Opioid use, prescribing patterns, and disposal after surgical procedures

**DOI:** 10.1016/j.rcsop.2024.100476

**Published:** 2024-07-14

**Authors:** Lauryn B. Hanson, Porter S. Hummel, Jacob W. Kokko-Ludemann, Kristi Lee, Linnea A. Polgreen

**Affiliations:** Department of Pharmacy Practice and Science, University of Iowa, 180 S. Grant Ave., Iowa City, IA 52242, USA

## 1 Background

Although effective for pain control, opioids have adverse effects that can be serious and life threatening, including the potential for physical dependence, overdose, and abuse.^1^ Recently, opioids have seen a decrease in prescribing^2^ due to advancing knowledge of pain management, changes in opioid prescribing legislation and guidelines, and the ongoing opioid epidemic.^3^ However, the opioid epidemic has persisted despite decreasing opioid prescribing trends. The result is an increase in diagnoses of substance-use disorders and opioid-overdose deaths.^4^ Indeed, opioid-related overdose deaths increased from 49,860 in 2019 to 81,806 in 2022.^5^ The most alarming increase has been in adolescents and young adults in the U.S. who primarily obtain their first opioids from the prescriptions of family members.^6^

There is a wide range in the amount and type of opioids being prescribed, even within specific types of surgical procedures, and these inconsistencies lead to excessive prescribing in some instances.^7^ Indeed, postoperative surveys indicate that the need for opioids after surgery is notably less than the amount prescribed.^8^ In a 2018 review, the amount of opioids prescribed was two to five times greater than the amount consumed.^9^ Many patients stop using opioids when they achieve adequate pain relief or if they experience intolerable side effects. Also, many patients who are prescribed opioids fill the prescription but do not take the opioids.^10^ The persistence of overprescribing leads to an accumulation of excess opioids in households.^8^

Recently, many prescribers have changed the way they treat pain after surgery to limit residual opioids after pain has been relieved.^11^ Most surgeons have reported that the opioid epidemic has significantly impacted their practice, yet the percentage of surgeons that counsel their patients on what to do with leftover opioids is remarkably low.^12^ This leads to the potential of leftover opioids being exposed to other members of the household and an increased risk of opioid abuse and overdose.^13^ There are more overdoses in those who have family members with prescription opioids than those who have family members with no prescription opioids.^13^ Risk for abuse and overdose increases with larger doses, longer-acting formulations, and more recent dispensing of a prescription to a family member.^14^ In addition, most patients also do not keep unused opioids in locked containers.^15^ Thus, easily accessible, leftover opioids can potentially contribute to the increased risk of overdose and development of a substance use disorder within members of a patient's family.

The persistence of the opioid epidemic means that actions of regulators, payers, and prescribers are not enough. Proper use, storage, and disposal of opioids is required. This study aims to collect knowledge of patient opioid use and what happens to opioids that go unused for participants with recent surgery.

## 2 Methods

Participant information and participation was gathered via an electronic survey sent out within a mass email to University of Iowa faculty, staff, and students. This study was approved by the University Institutional Review Board.

The survey was created and distributed using RedCap. The screening form and survey are included as supplemental material. Screening questions were asked to help determine eligibility to participate in the study. Participants were included in this study if they were over 18 years old, English speaking, able to provide consent, had surgery in the past 6 months, and were prescribed opioids postoperatively. The type of surgery was not limited. There were no further exclusion criteria.

Information gathered during the survey addressed postoperative outpatient opioid use and surgical history. Questions asked about medication use included information about the type of medication prescribed, how much medication was prescribed, how much medication was taken by the participant to control pain, what the participant did with any leftover medication and how it was stored, and the participant's plan for disposal of the medication. Participants were asked how soon after surgery the participant stopped taking their medication and the reason for stopping. Additional questions about the respondent's household demographics included how many adults and children live in the home with the participant.

The information gathered from eligible survey participants was compiled and summarized by surgery type. Surgery responses were categorized by type and anatomical location. Surgery categories include Abdominal (cholecystectomy, appendectomy, gallbladder removal, gastrectomy), Cancer-Related (lumpectomy, mastectomy, prostatectomy), Cosmetic (breast reduction), Dental/Oral (wisdom teeth removal, other oral surgery), Obstetric (cesarean section, hysterectomy), Ophthalmologic (laser vision correction), Orofacial (septoplasty, tonsillectomy), Orthopedic (rotator cuff surgery, joint replacement, broken bone repair, carpal tunnel), and Podiatric (bunionectomy, metatarsal realignment). Descriptive statistics, such as mean and standard deviation were used to describe opioid use by surgery type.

## 3 Results

**Fig. 1:**
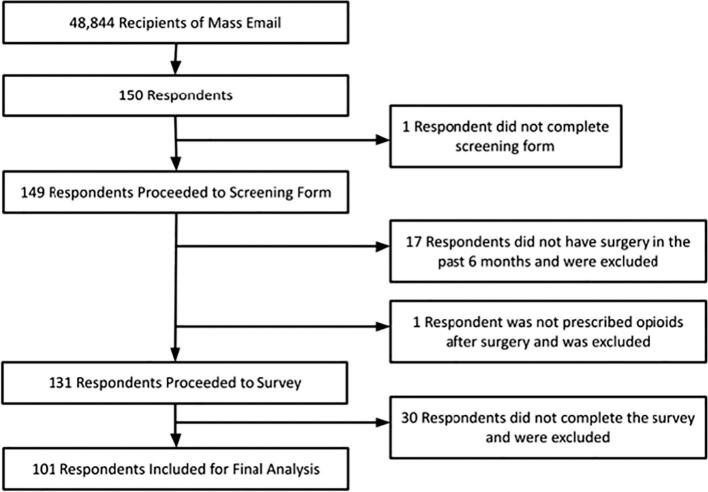
Distribution and recruitment of survey respondents.

The completed survey was distributed to a total of 48,844 recipients via email. Of those that received the email, 150 responded and proceeded to the screening form. A total of 49 participants were excluded. The final number of recipients included for analysis was 101 (Fig. 1).

Types of surgeries reported included dental, orthopedic, cesarean sections, and those requiring organs to be removed (gallbladder, tonsils, kidney). The average amount of opioids prescribed was 21 ± 18 tablets. Oxycodone and Hydrocodone were the most frequently prescribed, with some receiving Tramadol, Hydromorphone, or Morphine. Summary of opioid prescribing by surgery type is illustrated in Table 1. The most common surgery types reported were orthopedic/podiatric and dental/oral surgery, reported by 31.7% and 20.8% of participants respectively. The types of surgery that prescribed the most opioids on average were obstetric and orthopedic surgery.

**Table 1:** Numbers and types of opioids for surgery categories.

Opioid Average # tablets (# participants)	Abdominal	Cancer-related	Dental	Obstetric	Orthopedic/Podiatric	Other⁎⁎
Hydrocodone	23 (2)	11 (2)	11 (11)	25 (2)	21.7 (6)	15.3 (3)
Oxycodone	16.2 (10)	11.7 (9)	8.3 (10)	20 (5)	32.1 (27)	17.4 (5)
Other⁎	10.7 (3)	0	0	0	53 (5)	30 (1)
All	16 (15)	11.5 (11)	9.7 (21)	21.4 (7)	33.2 (38)	19.4 (9)

⁎ Includes tramadol, hydromorphone, morphine, codeine.

⁎⁎ Includes ophthalmological, orofacial, and cosmetic surgeries and a vasectomy.

Of the 101 participants, 31 (31%) finished the entire course of their regimen, 57 (56%) reported taking only some of their medication, and 13 (13%) did not take any of the opioids they were prescribed. Participants who stopped taking the medication early, reported that they stopped because their pain was controlled. Others chose to take another pain reliever such as Acetaminophen or Ibuprofen because it controlled their pain or because of the potential adverse effects of opioids. Of those who only took part of their regimen, 46% kept their leftover opioids to be used for future serious pain, while only 24% used a drug-take-back program or disposed of their opioids at home using a pharmacy-directed method. Others flushed them down the toilet (5%) or threw them in the trash (7%) Regarding participants' reported storage of opioids, most (56%) stored them in a secluded area such as a medicine cabinet, but 44% of participants stored their opioids in an accessible area such as the kitchen table, desk, nightstand, in a backpack or purse. Only 3 responded that the opioids were in a locked cabinet, and one responded that they were kept “out of reach”.

When asked about thoughts on how to minimize the number of opioids in the household, responses varied. Many participants endorsed support for limiting the prescribing of opioids after surgery to minimize the amount of leftover medication. Those who reported not taking any opioids after surgery endorsed increased prescribing of non-opioid pain regimens after surgery. Other suggestions included, “use natural pain relief like essential oils” and “flush them down that sink”. Lastly, most participants were enthusiastic about drug take-back programs, but many were unaware of these programs.

In terms of households, many participants were young adults: the largest age group is 20–30 years, but participants ranged in age from 18 to 81. Most participants lived with others. Only 9 (9%) lived alone. 54 (53%) participants lived with one other adult, and 74 (73%) had no children living in the same household. However, among those participants with children, 4 were babies, 5 were preschoolers, 14 were grade-school age, and 22 were teenagers. (See Table 2.)

**Table 2:** Participant demographics.

Variable	Average/Number
Age	41
Sex	
Male	26
Female	75
Other adults in the household	
0	13
1	54
2	27
3	4
4	3
Children in the household	
0	74
1	14
2	8
3	4
4	1
How much of the opioid prescription was taken	
All	31
Some	57
None	13

## 4 Discussion

This study helps confirm that there are many opportunities for opioid diversion in the household. Most participants only used a portion of their prescribed opioid regimen due to adequate pain control with other nonprescription medications, intolerable side effects experienced, or fear of severe side effects if they continued. Keeping leftover opioids in locked cabinets was uncommon (3%), and only 24% used a pharmacy-directed disposal method such as a disposal bag or drug-take-back program for leftover opioids. Other estimates of leftover-opioid disposal have been varied, ranging from 4.1 to 68.8%.^16^, ^17^, ^18^, ^19^

Opioid usage responses indicate that a majority of participants only used some of their prescribed regimen. Due to the “as needed” nature of most post-operational opioids, a regimen that is only partly used is not surprising. Adverse effects or the fear of adverse effects was listed as the main reason for early discontinuation or not taking any prescribed opioid at all. The adverse effects associated with opioids can be common, and often unbearable, especially constipation.^20^ Constipation was listed as the most common adverse effect of opioids taken and the adverse effect that posed the greatest barrier to initiation of opioid use. Fear of adverse events could lead to undertreated pain. However, a majority of participants believed their pain was not severe enough to warrant opioid use or had a strong opinion against opioid use. Of those that stopped their opioid regimens early or did not take any of their prescribed regimens, most indicated that their pain was adequately managed with nonprescription pain relievers. This poses a potential opportunity for intervention in the postoperative setting. Taking patient beliefs into account when prescribing a postoperative pain regimen is likely the best method to reduce unnecessary opioid prescriptions.

Having a remainder of the regimen saved as well as potentially having the medication accessible in an open area may result in unintended opioid adverse events for others within the household. Indeed, substance abuse happens with other adults and teenagers in the household.^13^^,^^21^ Most of the participants lived with other adults, and among those with children, teenagers were the most common age of children. However, household-opioid-related overdoses are also common among toddlers and preschoolers,^14^^,^^22^ and there were families in this study with children of that age as well. Most participants kept the opioids in cabinets, but cabinets are still accessible to many children and all household teens and adults.

Clearly, many participants had excess opioids: participants received an average of 30 pills, but 54.4% took less than the amount prescribed, and 12.9% took none. Previous research has reported similar results, with the percentage of patients with leftover opioids ranging from 41 to 64%.^16^^,^^23^^,^^24^ The percentages of patients that take none of their prescription have been reported from 14 to 20%.^16^^,^^23^^,^^24^ Prescribers could write fewer prescriptions or prescribe fewer pills with each prescription, but this could lead to undertreatment of pain. Pharmacists could also initially only partially fill prescriptions: those who use none or a few tablets will have fewer left over, but the full prescription is available to those who need it.^25^ In addition, prescribers could also encourage proper disposal or drug take-back programs at initial and follow-up appointments. Pharmacists and pharmacy techs could inform patients of locations participating in drug take-back programs.^26^ They could also provide adequate counseling on proper disposal of unwanted medications. Many participants were unaware of these disposal and drug-take-back options, but giving disposal kits to patients has been associated with decreases in opioids in the home.^17^

### 4.1 Limitations

This study was a pilot study and only aimed to investigate how opioids are used after surgery. We only interviewed 101 participants and thus the results might not be generalizable. A time frame of 6 months was chosen to include responses that were as accurate as possible, but it is possible that some responses are inaccurate due to poor recall of medications used after surgery. Also, Social-desirability bias is often a problem with surveys. This study serves as a framework for future studies to expand upon and investigate further.

## 5 Conclusions

Our results highlight the need for interventions to increase awareness of disposal programs in order to prevention diversion of medications. Future studies will be needed to expand upon these findings and identify potential harms that could result. In addition, our results highlight the need for interventions to increase appropriate opioid use, such as considering patient beliefs and perceptions of side effects.

## Funding

This work was supported in part by a grant from the National Center for Advancing Translational Science #UM1TR004403. NCATS had no role in the study design, collection, analysis and interpretation of data, writing of the report and the decision to submit it for publication.

## CRediT authorship contribution statement

**Lauryn B. Hanson:** Writing – review & editing, Writing – original draft, Visualization, Methodology, Investigation, Data curation, Conceptualization. **Porter S. Hummel:** Writing – review & editing, Writing – original draft, Visualization, Methodology, Investigation, Data curation, Conceptualization. **Jacob W. Kokko-Ludemann:** Writing – review & editing, Writing – original draft, Visualization, Methodology, Investigation, Data curation, Conceptualization. **Kristi Lee:** Writing – review & editing, Writing – original draft, Visualization, Methodology, Investigation, Data curation, Conceptualization. **Linnea A. Polgreen:** Writing – review & editing, Writing – original draft, Supervision, Methodology, Investigation, Funding acquisition, Data curation, Conceptualization.

## Declaration of competing interest

Linnea A. Polgreen has grant funding from the National Institutes of Health, and she is also a paid reviewer for the National Institutes of Health. The current work is unrelated to this funding. No other authors have any competing interests.

## Appendix A Supplementary data

Supplementary material 1: Screening Survey Supplementary material 1

Supplementary material 2: Main Survey Supplementary material 2
