# Supplementary material for: Opioid use, prescribing patterns, and disposal after surgical procedures
Source: Explor Res Clin Soc Pharm. 2024 Jul 14;15:100476. doi: 10.1016/j.rcsop.2024.100476 (PMC11662278; doi:10.1016/j.rcsop.2024.100476)
Supplement: Supplementary material 1: Screening Survey [file mmc1.pdf]

Screening Form

Please complete the survey below.

Thank you!

---

1) What is your age in years?

____

---

2) Have you had surgery in the last 6 months? This could be basic as bone, dental, c-section, appendectomy, knee replacement, joint replacement, etc.

◯ Yes
◯ No

---

3) Were you prescribed an opioid after surgery? Examples of opioids include Oxycodone (Oxycontin), Hydrocodone (Vicodin), Morphine (MS Contin), Tramadol (Ultram). etc.

◯ Yes
◯ No
