# Supplementary material for: Opioid use, prescribing patterns, and disposal after surgical procedures
Source: Explor Res Clin Soc Pharm. 2024 Jul 14;15:100476. doi: 10.1016/j.rcsop.2024.100476 (PMC11662278; doi:10.1016/j.rcsop.2024.100476)
Supplement: Supplementary material 2: Main Survey [file mmc2.pdf]

Survey

Please complete the survey below.

Thank you!

What was your assigned gender at birth?	◯ Male<br>◯ Female
What kind of surgery did you receive in the past 6 months?	__________________________________
What opioid medication did you receive? (ex. Oxycodone, Morphine, Hydrocodone)	__________________________________
What strength did you receive? (ex. 5mg, 10mg, etc.)	__________________________________
How many tablets did you receive?	__________________________________
For how many days were you supposed to take the medication?	__________________________________
How much of the medication did you take?	◯ All<br>◯ Some<br>◯ None
Why did you not take any of the medication?	__________________________________
For how many days did you take the medication?	__________________________________
How soon after the surgery did you start taking the medication?	__________________________________
Why did you stop taking the medication early?	__________________________________
Where did you store the medication?	__________________________________
What did you do with the leftover medication, if any?	__________________________________
How many adults live in your household besides yourself?	__________________________________
What are the ages of the adults?	__________________________________
How many children live in the household?	__________________________________

What are the ages of the children?

____

What ideas you do have to help minimize the number of opioids within households?

____

Would you like to be compensated for your involvement? (You will need to provide your name and address so that the compensation will be mailed to you. It may take at least 4 weeks for the compensation to arrive.)

◯ Yes
◯ No

What is your name?

____

What is your street address?

____

What is your city/town?

____

Which state?

____

What is the zipcode?

____
